# Household Food Insecurity and Women's Dietary Diversity in Seqota Declaration Pilot Woredas Across the Tekeze River Basin of Amhara and Tigray Regions

**DOI:** 10.1002/fsn3.70633

**Published:** 2025-07-18

**Authors:** Andinet Abera Hailu, Stephen Thornhill, Edward Lahiff

**Affiliations:** ^1^ Ethiopian Public Health Institute Addis Ababa Ethiopia; ^2^ University College Cork Cork Ireland

**Keywords:** agriculture, dietary diversity, Ethiopia, food insecurity, Seqota declaration

## Abstract

Food insecurity and inadequate dietary diversity worsen undernutrition in Ethiopia, hindering progress toward the Sustainable Development Goals and the Seqota Declaration's objective to eliminate hunger and chronic undernutrition by 2030. This study investigates the prevalence and causes of household food insecurity and dietary diversity among pregnant and lactating women (PLW) in the Seqota Declaration pilot woredas of the Tekeze River basin, situated in the Amhara and Tigray regions. A cross‐sectional survey conducted from March to April 2018 sampled 2036 households across 13 Seqota Declaration woredas. Food insecurity was assessed using the Household Food Insecurity Access Scale (HFIAS) and the Months of Adequate Household Food Provisioning (MAHFP) scale. Women's Dietary Diversity Score (WDDS) was evaluated for 642 PLW. Logistic and Poisson regression models identified predictors of food insecurity and dietary diversity, respectively. More than half of households in Tigray (55.0%) and Amhara (59.5%) experienced moderate to severe food insecurity. Only 7% of pregnant and lactating women (PLW) in Tigray and 10% in Amhara met the minimum dietary diversity requirements. Older household heads and those with lower wealth status faced a higher risk of food insecurity. Increased livestock ownership, improved land and water management practices, and greater production diversity were linked to lower odds of food insecurity. A larger land size (adjusted incidence rate ratio (AIRR) 1.04 per hectare, *p* = 0.017) and higher wealth status (AIRR 1.19 for the wealthiest quintile (Q5), *p* = 0.043; AIRR 1.17 for Q4, *p* = 0.044) improved Women's Dietary Diversity Score (WDDS). Seqota Declaration interventions should prioritize asset building, crop diversification, and improved land and water management to increase food security and dietary diversity. More research is needed to determine how market food availability, affordability, and recent events, such as conflicts and droughts, impact these predictors.

## Graphical abstract

In the Seqota Declaration pilot woredas, over half of households faced moderate to severe food insecurity, with only 7%–10% of pregnant and lactating women meeting minimum dietary diversity requirements. Older household heads and those with lower wealth status were at a higher risk of food insecurity. In contrast, increased livestock ownership, improved land and water management, and greater production diversity reduced these risks. Larger land size and higher wealth status significantly improved Women's Dietary Diversity Score.

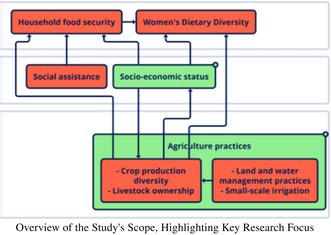

**Abbreviations**
AIRR: Adjusted incidence rate ratio
AOR: Adjusted odds ratio
CI: Confidence interval
ENGINE: Empowering New Generations to Improve Nutrition and Economic Opportunities
EPHI: Ethiopian Public Health Institute
FANTA: Food and Nutrition Technical Assistance
FAO: Food And Agriculture Organization
HFIAS: Household Food Insecurity Access Scale
HH: Household
HLPE: High‐Level Panel of Experts on Food Security and Nutrition
IFAD: International Fund for Agricultural Development
IRR: Incidence rate ratio
MAHFP: Months of Adequate Household Food Provisioning
MDD‐W: Minimum Dietary Diversity for Women
MIYCN: Maternal, Infant, and Young Child Nutrition
NGO: Non‐Governmental Organization
ODK: Open Data Kit
OR: Odds ratio
PCA: Principal component analysis
PLW: Pregnant and lactating women
PPS: Probability proportionate to population
SBCC: Social and behavior change communication
SD: Seqota Declaration
SDev: Standard deviation
SDG: Sustainable Development Goal
TLU: Tropical Livestock Unit
UNICEF: United Nations International Children's Emergency Fund
WDDS: Women's Diet Diversity Score
WFP: World Food Programme
WHO: World Health Organization

## 1 Background

The primary goal of Sustainable Development Goal 2 (SDG 2) is to eradicate hunger and malnutrition by 2030 (United Nations 2015). However, since 2014, food insecurity has risen globally, exacerbated by factors such as the COVID‐19 pandemic, income inequality, conflict, and climate change (FAO et al. 2024). A significant nutritional crisis is evident in Ethiopia, where 52.1% of the population experienced moderate to severe food insecurity in 2019 (Telila and Sima 2024). Additionally, only 7% of women aged 15 to 49 achieved minimum dietary diversity, defined as consuming five or more food groups (Ethiopian Public Health Institute (EPHI) 2023).

The Seqota Declaration (SD), Ethiopia's ambitious initiative to eliminate hunger and chronic undernutrition by 2030, targets 33 pilot woredas in the drought‐prone Tekeze River basin across Amhara and Tigray, where erratic rainfall, land degradation, and rocky terrain limit agricultural productivity (The Federal Democratic Republic of Ethiopia Seqota Declaration 2016). Characterized by a short rainy season (July to August) and poor agricultural productivity, these regions face chronic food shortages and were therefore selected as pilot areas for the first phase of the government‐led SD interventions. The SD initiative is being implemented in three phases, with the final phase set to conclude in 2030. The first phase focuses on specific woredas within the Tekeze River Basin. The second phase, scheduled for 2021–2026, aims to expand into additional regions. The third phase seeks to achieve nationwide scalability by leveraging the successful interventions and insights obtained from earlier stages.

The SD's first phase (2018–2020) implemented nutrition‐specific, nutrition‐sensitive, and infrastructure interventions across the health, agriculture, water, education, and social protection sectors to address food insecurity and improve dietary diversity, particularly for vulnerable groups, including pregnant and lactating women (PLW). Food insecurity and poor dietary diversity are driven by various factors, including socioeconomic status (Harris‐Fry et al. 2015), food availability (FAO et al. 2019), agricultural production diversity (Jones et al. 2014), access to land (Deininger and Byerlee 2011), and soil management practices (Heng et al. 2014). Agricultural practices, such as livestock ownership and crop diversification, are essential for building resilience by providing income and creating a buffer against shocks, while diverse crops enhance food access stability (Jones et al. 2014; Megersa et al. 2014; Sibhatu and Qaim 2018). The heavy reliance on staple crops, such as cereals, and the limited production of nutrient‐rich foods, including vegetables, continue to constrain dietary diversity (Hirvonen 2019a, 2019b; Hirvonen and Hoddinott 2017). Low dietary diversity poses risks to maternal and child health, as a lack of nutrient‐rich foods, such as fruits, vegetables, and animal‐source foods, leads to nutrient inadequacy (Esaryk et al. 2021).

While the implementation of Seqota Declaration interventions follows a multi‐sectoral approach to address household food insecurity and maternal and child undernutrition, there remains a limited understanding of how structural factors (e.g., land ownership and productivity, irrigation practices, and production diversity) and behavioral factors (e.g., awareness of maternal nutrition messages) interact to influence household food security and dietary diversity, particularly for vulnerable groups like pregnant and lactating women. This study covered a wide range of multisectoral factors across the remote woredas of the Tekeze River Basin, which are often overlooked by existing studies. Addressing this research gap is critical for enhancing the effectiveness of Ethiopia's Seqota Declaration by identifying successful interventions for scaling up and hence achieving the goal of reducing nutritional vulnerability by 2030 nationwide.

Understanding the interplay between structural factors (e.g., agricultural practices such as crop diversification and livestock ownership) and behavioral factors can inform targeted interventions that enhance both household food security and dietary diversity, especially for PLW who are at a higher risk of malnutrition (High Level Panel of Experts on Food Security and Nutrition 2020). The research question guiding this study is how socioeconomic disparities, agricultural practices, and behavioral factors interact to influence household food insecurity and dietary diversity among households in SD pilot woredas of the Amhara and Tigray regions, and what the implications are for the future scaling up of the SD initiative at the national level. The study's findings can help policymakers and development practitioners design evidence‐based programs that address both the immediate and underlying causes of food insecurity in rural Ethiopia.

## 2 Methods

### 2.1 Study Area and Settings

The SD household survey was conducted between March and April 2018 in 13 woredas along the Tekeze River basin, which includes eight in the Amhara region and five in the Tigray region. The Tekeze River stretches over 600 km from its source in the central Ethiopian highlands of Lasta to the border with Sudan, maintaining an average elevation of 1850 m above sea level and covering a catchment area of approximately 68,000 km^2^. Approximately 70% of the basin comprises highlands with elevations exceeding 1500 m. The upper sections of the Tekeze are bordered by mountains rising above 2000 m, with the highest peak of the Ras Dashan system reaching 4620 m above sea level (The Federal Democratic Republic of Ethiopia Seqota Declaration 2016). The woredas in the SD intervention phase are situated in food‐insecure areas of the Tekeze River basin, characterized by rugged terrain, hills, gorges, and barren land with rocky soil. Agriculture in these woredas primarily relies on the 2‐month rainy season that occurs between July and August (Awlachew et al. 2008).

### 2.2 Study Design

The study employed a cross‐sectional household survey design to capture a snapshot of food insecurity and dietary diversity (Rothman et al. 2008). A structured questionnaire was used to establish a baseline for the Seqota Declaration (SD) innovation phase interventions (The Federal Democratic Republic of Ethiopia 2016). The questionnaire focused on key performance indicators related to essential nutrition‐sensitive, nutrition‐specific, and socioeconomic characteristics of households.

### 2.3 Sampling Strategy

Thirteen woredas—five in Tigray and eight in Amhara (Table 1)—were purposefully selected for the baseline survey of the Seqota Declaration (SD) intervention based on available resources and the criteria established by the evaluation team, with final approval from the SD implementation team. The evaluation aimed to identify a successful approach for scaling up effective interventions during SD Phase 1 by prioritizing woredas with a strong potential to implement key nutrition‐specific and nutrition‐sensitive interventions. The criteria for selecting woredas included: (1) the existence of woreda‐specific plans and interventions under the SD framework, along with a readiness to initiate interventions immediately after the baseline; (2) inclusion in the Productive Safety Net Program (PSNP); (3) the presence of at least one major NGO‐supported nutrition or food security program; and (4) a relatively large population size (White and Sabarwal 2014).

**TABLE 1:** Socio‐demographic and agricultural characteristics of the sample households.

Variables (*N* = 2036)	Frequency	Percent	95% lower	95% upper
Region				
Tigray	1108	54.4	44.0	64.5
Amhara	928	45.6	35.5	56
The religion of the HH head				
Orthodox	1993	97.9	93.1	99.4
Muslim	43	2.1	24.10	36.50
Gender of HH head				
Male	1725	84.7	82.3	86.9
Female	311	15.3	13.1	17.7
Age of household head	2036	47.7	46.7	48.6
Marital status of HH head				
Single	26	1.3	0.9	2.0
Married	1679	82.5	80.2	84.6
Living together	2	0.1	0.0	0.7
Divorced	123	6.0	4.7	7.6
Separated	17	0.8	0.5	1.5
Widowed	189	9.3	7.8	11.0
Household head education				
No formal education	1466	72.0	68.8	74.9
1–8 years	507	24.9	22.1	27.9
Above high school	63	3.1	2.2	4.4
Household main livelihood				
Self‐produced crops	1919	94.3	92.5	95.6
Daily labor	39	1.9	1.1	3.4
Employment wage	13	0.7	0.3	1.3
Remittance and other supports	65	3.2	2.38	4.21
Wealth quintiles				
Poorest	551	27.1	23.1	31.4
Second	513	25.2	22.4	28.3
Middle	398	19.6	17.5	21.8
Fourth	353	17.3	14.5	20.6
Richest	221	10.8	8.48	13.7
Any cash or food assistance received in the past year				
No	1257	61.7	55.8	67.4
Yes	779	38.3	32.6	44.2
Livestock (Oxen, Cow, Heifer) owned by HH				
None	350	17.2	15.0	19.5
1–5	1586	77.9	75.2	80.4
6+	100	4.9	3.4	7.3
Tropical Livestock Unit (TLU)		2.50	2.31	2.67
Hectares of agricultural land accessed (Ha)				
< 0.5	849	41.7	37.1	46.5
0.5–2.0	1100	54.0	49.8	58.1
> 2.0	87	4.3	3.2	5.7
Mean (SDev) in hectares		2.23	2.1	2.4
Land and water management practice				
No	408	20.0	17.0	23.4
Yes	1628	80.0	76.6	83.0
Small‐scale irrigation				
No	1768	86.8	81.5	90.8
Yes	268	13.2	9.22	18.5
Use of fertilizers				
No	208	10.2	7.7	13.5
Yes	1828	89.8	86.5	92.3

The sampling procedure involved three stages, and selected kebeles (the smallest administrative unit) and gotes (the minor geographic division within a kebele) were selected based on their population size. A list of kebeles and gotes was collected from the respective woreda administrative offices in each woreda. In the first stage, seven kebeles were chosen using probability proportionate to population size (PPS) in each of the 13 woredas (Tyrer and Heyman 2016). In each kebele, a list of gotes was obtained from the kebele administrations, where the data collection supervisors employed a lottery method to select one gote. Lastly, all households residing in the selected gote were listed to establish a sampling frame for the final stage of household selection. A systematic random sampling technique was used to select 25 families from each group for the interview.

### 2.4 Sample Size Determination

The sample size was determined based on the regional‐level representation, which combined the selected SD woredas in Amhara and Tigray for critical outcomes and intervention coverage indicators. The study was not designed to provide comparable estimates across woredas—this sample size aimed to detect differences between regions within the SD framework in the change of food security status. The anticipated level of change in the outcome indicators for the sample size calculation was grounded in findings from the multi‐sectoral ENGINE project evaluation in the Amhara region (U.S. Agency for International Development 2016). The aim was to achieve roughly equal sample sizes in the two areas. However, due to the differences in the prevalence of indicators used for sample size calculation, the required sample size was slightly higher in Tigray. A total of 2036 households were surveyed in both regions. The sample size needed was computed based on the equations described elsewhere (Heeringa 2012):

(1) $$N=\text{deff}\times {\left(\frac{Z\partial }{2}+\mathrm{z\beta}\right)}^{2}\times 2\times p\times q/\left(d^{2}\right)$$

where *p* = (*p*1 + *p*2)/2 and *q* = (*q*1 + *q*2)/2, *q*1 = (1 − *p*1) and *q*2 = (1 − *p*2), *N* = required minimum sample size, *p*1 = prevalence of food insecurity at baseline, *p*2 = expected food insecurity at end‐line, *d* = size of change desired to detect (*p*2 − *p*1), *zα*/2 = *z*‐score corresponding to the degree of confidence with which it is desired to conclude that an observed change of magnitude (*p*2 − *p*1) would not have occurred by chance, *α*/2—the level of statistical significance for a two‐tailed test at a 95% confidence level and 1.96 standard deviations, *zβ* = the *z*‐score corresponding to the degree of confidence with which it is desired to be sure of detecting a change of magnitude (*p*2 − *p*1) if one occurred, *β* = statistical power of 80%, deff = design effect.

### 2.5 Survey Tools, Data Collection, and Quality Assurance

The survey questionnaire was designed based on the SD key performance indicators to measure constructs, including socio‐demographic, economic, dietary patterns, food security, agriculture, and other livelihood and infrastructure variables. The questionnaire modules were prepared in English, translated into Amharic and Tigrigna, and then retranslated into English to ensure consistency. The questionnaire was uploaded to the Open Data Kit (ODK) application in all three languages (Madon et al. 2023).

Data were collected across 13 evaluation woredas in the Amhara and Tigray regions by 92 independent enumerators and 14 field supervisors, specifically hired for this study on a 2‐month contract. The enumerators were carefully selected to ensure reliability and reduce bias; most had prior experience in data collection, which ensured their familiarity with survey methodologies, while a few were recent graduates with relevant academic backgrounds in public health or social sciences. Enumerators and supervisors participated in an intensive 3‐week training program covering the survey's objectives, sampling procedures, interview techniques, the contents of the questionnaire modules, and the Open Data Kit (ODK) application. The ODK system was designed to enhance data consistency through built‐in quality assurance checks, including cross‐checking questions, logic for automatically skipping non‐applicable questions, and prompts tailored to specific gender and age groups. Data were transferred daily to the Ethiopian Public Health Institute's server in Addis Ababa immediately after completing each household survey, enabling real‐time data monitoring. The data manager reviewed the data daily and provided feedback to the field team before they left the data collection sites, allowing enumerators to correct errors promptly. Additionally, team supervisors conducted random re‐interviews on 2% of households in each enumeration area to ensure enumerator reliability, focusing on selected indicators to verify data accuracy. Backup data was also stored on an external disk to prevent data loss.

### 2.6 Pretesting of the Survey Tools and Field Implementation

The survey teams tested the electronic questionnaires in a non‐study area, refining them before deployment. The pretest exercise, conducted on 5% of the sample, assessed how the data collection team introduced the tools to respondents and how respondents interpreted the questions. This pretest enabled the research team to understand practical scenarios and make final device edits before actual fieldwork. The officers in charge of specific areas assisted in obtaining authorization letters to access the study locations. The supervisors overseeing the fieldwork secured permission letters from the local administrations. After gaining entry, supervisors provided information about the study to health and agriculture extension workers in each kebele, who were responsible for their respective communities.

### 2.7 Dependent and Independent Variables

The dependent variables in this study are the household food insecurity access score (Coates et al. 2007) and women's dietary diversity score (Food and Agriculture Organization of the United Nations (FAO) 2016), both of which are critical indicators of nutritional vulnerability within the framework of Ethiopia's Seqota Declaration. Household food insecurity status was assessed using the Household Food Insecurity Access Scale (HFIAS), a widely validated tool developed by the Food and Nutrition Technical Assistance (FANTA) project. The HFIAS consists of nine occurrence and frequency‐of‐occurrence questions that measure the severity of food insecurity over the past 30 days, focusing on three domains: anxiety and uncertainty regarding food supply, insufficient quality of food, and inadequate food intake, along with its physical consequences (Coates et al. 2007). Responses to the HFIAS questions were analyzed to obtain a continuous score ranging from 0 to 27, calculated by summing the frequency‐of‐occurrence scores for each household, with higher scores indicating greater food insecurity, following the methodology outlined by Coates et al. (2007). The score was then categorized into food insecurity prevalence, which includes food secure, mild food insecure, moderately food insecure, and severely food insecure groups, which were further categorized into two groups—food secure (food secure and mildly food insecure) and food insecure (moderately food insecure and severely insecure) for the binary logistic regression analysis.

To complement the HFIAS, the Monthly Adequate Household Food Provisioning (MAHFP) was calculated to assess the duration of adequate food access over the past 12 months, providing a longitudinal perspective on food security. Following the methodology outlined by the FANTA project, the MAHFP was measured by asking households to recall the months in the past year when they experienced food shortages (Bilinsky and Swindale 2010). The MAHFP score was calculated as the total number of months (ranging from 0 to 12) in which the household reported adequate food provisioning, with higher scores indicating greater stability in food security. This metric enhances the assessment of household food insecurity by capturing seasonal variations and chronic challenges to food access, supporting the Seqota Declaration's aim to address nutritional vulnerability through targeted interventions.

The second dependent variable, women's dietary diversity score (WDDS), was designed to assess the dietary quality of women of reproductive age (15–49 years) within the sampled households. The WDDS was calculated according to the Food and Agriculture Organization's (FAO) guidelines for measuring individual dietary diversity, which included a 24‐h dietary recall to evaluate the consumption of ten food groups: grains, white roots and tubers, legumes, nuts and seeds, dairy, meat/poultry/fish, eggs, dark green leafy vegetables, vitamin A‐rich fruits and vegetables, and other fruits and vegetables (Food and Agriculture Organization of the United Nations (FAO) 2016). Each food group consumed received a score of 1, resulting in a total WDDS that ranged from 0 to 10, with higher scores indicating greater dietary diversity and improved nutritional intake (Food and Agriculture Organization of the United Nations (FAO) 2016). This method, widely utilized in nutrition studies, is a proxy for micronutrient adequacy among women, a vulnerable group targeted by the Seqota Declaration (Arimond et al. 2010).

The independent variables in this study were selected based on a conceptual framework that identifies agricultural and socioeconomic factors as key determinants of household food insecurity and women's dietary diversity within Ethiopia, as outlined in the Seqota Declaration. Agricultural practices were comprehensively assessed through various variables capturing crop cultivation, livestock ownership, land use, and farming inputs, reflecting the multifaceted nature of agricultural production in the study areas. Crop cultivation was evaluated as a set of binary variables indicating whether households cultivated specific crop types during the previous agricultural season, covering seven categories: cereals, legumes and nuts, seeds and oil crops, root and tuber crops, vegetables, fruits, and cash crops (Jones et al. 2014). Each crop type was coded as 1 if cultivated and 0 otherwise, providing insight into how crop production diversity influences food availability and dietary diversity across regions. The Tropical Livestock Unit (TLU) was calculated to quantify livestock ownership, a critical component of household food security in Ethiopia's mixed farming systems. The TLU was derived by aggregating the number of different animals owned by the household (e.g., cattle, sheep, goats, poultry) using standardized conversion factors: for instance, cattle = 1.0 TLU, sheep/goats = 0.1 TLU, and poultry = 0.01 TLU, following FAO guidelines (Food and Agriculture Organization of the United Nations 2018). The TLU captures household livestock ownership, which helps mitigate food insecurity by providing direct food sources and generating supplemental income.

The land size, measured in hectares, was included as a continuous variable to capture the extent of agricultural land available to each household, which is a key determinant of food production capacity (Devereux 2000). Land and water management practices were assessed as a binary variable, coded as 1 if the household implemented practices such as terracing, contour plowing, or water harvesting, and 0 otherwise. This reflects efforts to mitigate land degradation and improve soil moisture retention in the drought‐prone study areas (The Federal Democratic Republic of Ethiopia 2016). Small‐scale irrigation beneficiary status was recorded as a binary variable (1 = yes, 0 = no), indicating whether the household benefited from small‐scale irrigation schemes promoted under the Seqota Declaration to enhance agricultural productivity during dry spells. Using fertilizers and improved seeds and seedlings was each measured as a binary variable (1 = yes, 0 = no), capturing the adoption of modern agricultural inputs that can increase crop yields and resilience to environmental stressors. These agricultural variables provide a comprehensive picture of farming practices and their potential impact on household food security and women's dietary diversity.

Socio‐economic factors were also incorporated to account for household‐level characteristics that mediate food security outcomes. These factors include geographic location to capture regional variations and household wealth, measured using a wealth index constructed through principal component analysis (PCA) of household assets, in line with the methodology of the Demographic and Health Surveys (DHS) program (2004). Assets included in the PCA encompassed ownership of durable goods (e.g., radio, bicycle), housing characteristics (e.g., roof material), and access to services (e.g., electricity). To illustrate economic disparities, the resulting wealth index was categorized into quintiles, ranging from the poorest to the wealthiest. The education level of the household head was recorded as a categorical variable with three levels: no formal education, primary education, and secondary education or higher, reflecting human capital that may influence food security decision‐making.

**FIGURE 1:**
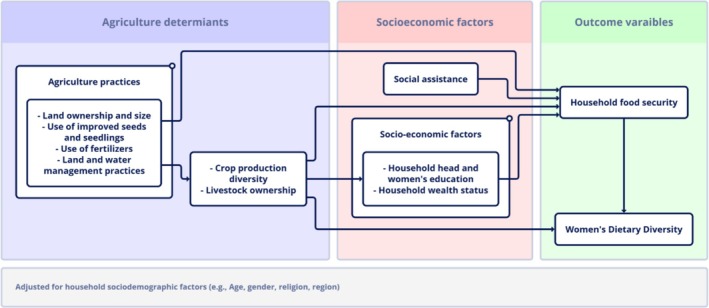
Conceptual framework illustrating the determinants of food security and women's dietary diversity in the study.

The selection of these independent variables aligns with the Seqota Declaration's emphasis on addressing food insecurity through multisectoral interventions that target regional disparities, agricultural productivity, socio‐economic barriers, and environmental challenges in Tigray and Amhara. Figure 1 illustrates the theoretical framework, showing how various agriculture‐related, socioeconomic, and demographic factors can impact household food security and women's dietary diversity. The theoretical relationships were statistically tested in conjunction with the food insecurity status of the study households and the women's dietary diversity score.

### 2.8 Statistical Models and Analytical Approach

Descriptive statistics summarized the prevalence of food insecurity, the Women Dietary Diversity Score (WDDS), and sample characteristics, presenting frequencies, percentages, and confidence intervals for categorical variables and means and standard deviations for continuous variables. All analyses accounted for the survey design by applying weights to adjust for unequal selection, clustering, and stratification probabilities (Ahmed 2024).

Different regression models were used to identify risk factors for food insecurity and determinants of WDDS, each following a three‐step process. A logistic regression model was used to analyze food insecurity. Initially, bivariate logistic regression was utilized to estimate unadjusted odds ratios (ORs) and 95% confidence intervals (CIs) for each independent variable. Variables with a *p*‐value of < 0.1 were considered for inclusion in the multivariable logistic regression (Jeffrey 2010).

A similar approach was followed for WDDS, but Poisson regression models were employed because of the count nature of the outcome (Hardin and Hilbe 2014). Bivariate Poisson regression was initially used to estimate unadjusted incidence rate ratios (IRRs) and 95% CIs for each independent variable considered relevant according to the conceptual framework. Subsequently, a multivariable Poisson regression model was developed, incorporating all variables with a *p*‐value of < 0.1. Exponentiated coefficients were presented as incidence rate ratios (IRRs) for bivariate models and adjusted incidence rate ratios (AIRRs) for multivariable models (Hardin and Hilbe 2014). Unless otherwise noted, the statistical significance of the final models was established at *p* < 0.05. Results were presented with odds ratios (ORs), adjusted odds ratios (AORs), IRRs, adjusted incidence rate ratios (AIRRs), 95% CIs, and exact *p*‐values, except for *p* < 0.001, which was reported as such. All analyses were conducted using Stata version 16 (StataCorp 2019).

### 2.9 Bivariate Analysis

The logistic bivariate analysis models the log‐odds of food insecurity (1/0) against each predictor variable individually (Equation 2), whereas the Poisson bivariate regression models the women's dietary diversity score (WDDS) (Equation 3).

(2) $$\text{logit}\;\left(P\left(Y_{i}=1\right)\right)=\ln\;\left(P\;\left(Y_{i}=1\right)/\left(1-P\left(Y_{i}=1\right)\right)\right)=\beta_{0}+\beta_{1}X_{\mathrm{ji}}$$

(3) $$\ln\;\left(\mu_{i}\right)=\beta_{0}+\beta_{1}X_{\mathrm{ji}}$$

where *Y*_*i*_: Binary food insecurity outcome for household *i*, *μ*_*i*_: Expected WDDS count for individual *i*, *X*_*ji*_: The *j* ^th^ predictor variable for household *i*, *β*_0_: Intercept, *β*1: Coefficient for the predictor *X*_*ji*_.

Equations (2) and (3) were applied separately for each of the predictor variables.

### 2.10 Multivariable Regression

The multivariable logistic regression models the log‐odds of the probability of food insecurity (binary: 1 = insecure, 0 = secure) (*P*(*Y*_*i*_ = 1)) for household i, using a logit function (Equation 4).

(4) $$\begin{array}{c} \text{logit}\;\left(P\left(Y_{i}=1\right)\right)=\ln\;\left(P\;\left(Y_{i}=1\right)/\left(1-P\left(Y_{i}=1\right)\right)\right)=\beta_{0}\\ \;+\beta_{1}{\text{Region}}_{i}+\beta_{2}{\mathrm{Sex}}_{i}+\beta_{3}{\text{Religion}}_{i}+\beta_{4}{\mathrm{Age}}_{i}\\ \;+\beta_{5}{\text{Education}}_{i}+\beta_{6}{\text{Wealth index}}_{i}+\beta_{8}{\text{Land size}}_{i}\\ \;+\beta_{9}\text{Cash}/{\text{food assistance}}_{i}+\beta_{10}\;{\mathrm{TLU}}_{i}\\ \;+\beta_{11}\text{land}/{\text{water managment}}_{i}+\beta_{12}{\text{Irrigation practice}}_{i}\\ \;+\beta_{13}\mathrm{Use}\;{\text{of fertilers}}_{i}+\beta_{14}{\text{Production diversity}}_{i}+{ℇ}_{i} \end{array}$$

where *Y*_*i*_: Binary outcome (1 = food insecure, 0 = food secure) for household *i*, *β*0: Intercept, *β*1–*β*14: Coefficients for each predictor variable. Independent variables were coded appropriately (e.g., Sex: 0 = male, 1 = female; Wealth Index: categorical).

The Poisson regression models the expected count of WDDS (a count of food groups consumed) for individual i, using a log function (Equation 5).

(5) $$\begin{array}{c} \ln\;\left(\mu_{i}\right)=\ln\;\left(P\;\left(Y_{i}=1\right)/\left(1-P\left(Y_{i}=1\right)\right)\right)=\beta_{0}\\ \;+\beta_{1}{\text{Region}}_{i}+\beta_{2}{\text{Wealth}}_{i}+\beta_{3}\text{Cash}/{\text{food assistance}}_{i}\\ \;+\beta_{4}\text{Exposure}\;{\text{to MIYCN}}_{i}+\beta_{5}\text{Food security}\;{\left(0/1\right)}_{i}\\ \;+\beta_{6}{\text{MAHFP}}_{i}+\beta_{7}{\text{Land size}}_{i}+\beta_{8}\;{\mathrm{TLU}}_{i}\\ \;+\beta_{9}\mathrm{Use}\;{\text{of fertilers}}_{i}+\beta_{10}{\text{Production diversity}}_{i}+{ℇ}_{i} \end{array}$$

For WDDS (count), the Poisson regression models the expected count, with coefficients exponentiated to yield Incidence Rate Ratios (IRRs) and Adjusted IRRs (AIRRs): Where *μ*_*i*_: Expected WDDS count for individual *i*, *β*0: Intercept, *β*1–*β*13: Coefficients for each predictor variable.

The final regression model was approved after considering issues such as multicollinearity, heteroskedasticity of residuals, and model modifications. The tested model specification did not pose any problems with the final model.

## 3 Results

### 3.1 Household Characteristics

Table 1 summarizes the socio‐demographic characteristics and agricultural practices of surveyed households. Households were primarily located in Tigray (54.4%) and Amhara (45.6%), with nearly all household heads identifying as Orthodox (97.9%) and a small proportion as Muslim (2.1%). Most household heads were male (84.7%), with an average age of 47.7 years. More than eight in ten household heads were married, followed by smaller proportions of individuals who were widowed, divorced, separated, cohabiting, or single.

Education levels indicated that 72.0% of household heads had no formal education, 24.9% had 1–8 years of education, and 3.1% had an education above high school. Self‐produced crops were the primary livelihood for 94.3% of households, with minor contributions from daily labor, employment wages, or remittances. Wealth distribution revealed that 27.1% of households fell into the poorest quintile, with the remaining households distributed across the second (25.2%), middle (19.6%), fourth (17.3%), and richest (10.8%) quintiles. Approximately 38.3% of households received cash or food assistance in the past one year.

Regarding agricultural practices, 77.9% of households owned 1–5 livestock animals (oxen, cows, and heifers), with an average of 2.23 animals and a mean Tropical Livestock Unit (TLU) of 2.5. Households' land access is limited, with 41.7% of households owning < 0.5 ha, 54.0% owning between 0.5 and 2.0 ha, and 4.3% owning more than 2.0 ha, averaging 2.23 ha of land ownership. Land and water management practices were adopted by 80.0% of households, 13.2% used small‐scale irrigation, and 89.8% applied fertilizers during the previous cropping year.

### 3.2 Household Food Security Status

Table 2 illustrates the distribution of households based on the HFIAS. More than half of the households (58.2%) reported consuming only a limited variety of foods, 52.8% were unable to eat their preferred foods, and 52.4% expressed concern about not having enough to eat. Additionally, 46.7% consumed foods they would rather not eat, 43.9% had smaller meals than necessary, and 39.6% ate fewer meals throughout the day. Under severe conditions, 14.1% reported having no food, 9.4% went to bed hungry, and 5.2% spent an entire day and night without eating.

**TABLE 2:** Household food insecurity access scale (HFIAS) conditions of surveyed households (*N* = 2036).

HAFIAS conditions	*N*	Percent	95% lower	95% upper
Worried about not having enough food	1067	52.4	48.2	56.5
Not able to eat the kinds of foods preferred	1075	52.8	48.7	56.9
Eaten just a few kinds of food	1185	58.2	54.2	62.1
Eaten food that I prefer not to eat	952	46.7	42.7	50.9
Eaten a smaller meal than needed	894	43.9	39.5	48.4
Eaten fewer meals in a day	806	39.6	35.1	44.2
No food of any kind in the household	287	14.1	11.4	17.3
Went to sleep at night while hungry	191	9.4	7.3	11.9
I spent a whole day and night without eating	105	5.2	3.78	7.03

### 3.3 Household Food Insecurity Prevalence

Table 3 presents a comparative analysis of food insecurity prevalence in Ethiopia's Tigray and Amhara regions, specifically in Seqota Declaration woredas, based on a sample of 2036 households. Food insecurity was assessed using the Household Food Insecurity Access Scale (HFIAS) and the Months of Adequate Household Food Provisioning (MAHFP).

**TABLE 3:** Food insecurity prevalence based on HFIAS/MAHFP scores by region (*n* = 2036).

Variables	Tigray (*n* = 1108)	Amhara (*n* = 928)	*p*
	*N* [%, 95% CI]	*N* [%, 95% CI]	
Food insecurity prevalence using HFIAS			
Food secure	375 [33.8%, 28.2–39.9]	271 [29.2%, 24.9–33.9]	0.5506
Mildly food insecure	124 [11.2%, 8.9–14.1]	105 [11.3%, 8.7–14.7]	
Moderate food insecure	392 [35.4%, 31.4–39.6]	350 [37.8%, 33.9–41.8]	
Severely food insecure	217 [19.6%, 15.3–24.7]	202 [21.7%, 17.3–26.9]	
Food insecurity prevalence using MAHFP			
Food secure	369 [33.3%, 28.8–38.2]	254 [27.4%, 23.5–31.5]	< 0.001
Mildly food insecure	247 [22.3%, 19.0–25.9]	330 [35.5%, 31.2–40.1]	
Moderate food insecure	339 [30.5%, 25.9–35.7]	263 [28.3%, 24.2–32.9]	
Severely food insecure	154 [13.9%, 10.3–18.4]	82 [8.8%, 6.5–11.9]	

*Note:* Food insecurity prevalence categories were assessed using the Household Food Insecurity Access Scale (HFIAS) and the Months of Adequate Household Food Provisioning (MAHFP) scale. The data include the sample size (*n*), percentage (%), and 95% confidence intervals (CI). The *p*‐value indicates the statistical significance of differences between regions, with a *p*‐value of < 0.05 suggesting statistically significant differences.

Using the HFIAS, the prevalence of food insecurity was comparable in both regions, with no statistically significant difference (*p* = 0.5506). In Tigray, 33.8% of households were food secure (95% CI 28.2–39.9), compared to 29.2% in Amhara (95% CI 24.9–33.9). Mild food insecurity was nearly equal, affecting 11.2% of households in Tigray and 11.3% in Amhara. Moderate food insecurity was the most common category in both regions, impacting 35.4% of households in Tigray and 37.8% in Amhara. Severe food insecurity was reported in 19.6% of Tigray households and 21.7% of Amhara households.

Using the MAHFP metric, significant differences were observed between the regions (*p* < 0.001). In Tigray, 33.3% of households were food secure (95% CI 28.8–38.2), compared to 27.4% in Amhara (95% CI 23.5–31.5). Mild food insecurity was notably higher in Amhara (35.5%, 95% CI 31.2–40.1) than in Tigray (22.3%, 95% CI 19.0–25.9). Moderate food insecurity was more common in Tigray (30.5%, 95% CI 25.9–35.7) than in Amhara (28.3%, 95% CI 24.2–32.9). However, severe food insecurity was more prevalent in Tigray (13.9%, 95% CI 10.3–18.4) than in Amhara (8.8%, 95% CI 6.5–11.9). These findings indicate regional variations in the duration and severity of food insecurity, with Amhara experiencing a higher prevalence of mild food insecurity. At the same time, Tigray displays a greater proportion of severely food‐insecure households.

**FIGURE 2:**
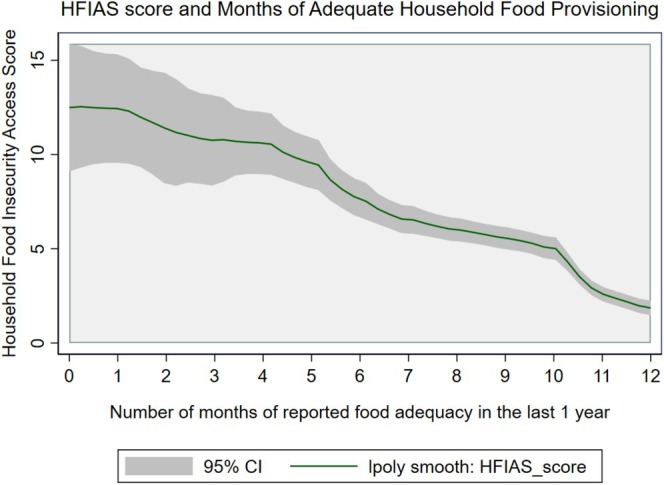
Local polynomial correlation between household food insecurity measures (Household Food Insecurity Access Scale (HFIAS) and Monthly Adequate Household Food Provisioning (MAHFP)). The green line represents the locally weighted scatterplot smoothing (LOESS) fit of the HFIAS score, while the gray shaded area denotes the 95% confidence interval.

Figure 2 presents a visualization of the relationship between the food insecurity metrics, namely the Household Food Insecurity Access Scale (HFIAS) score, which is based on thirty 30‐day recall period, and the number of months of adequate household food provisioning (MAHFP) over the previous year. The graph employs a local polynomial smoothing technique to depict the trend, with a green line representing the smoothed HFIAS score and a shaded gray area indicating the 95% CI, highlighting the inverse relationship between the HFIAS score and the duration of food adequacy. The data reveal a clear downward trend of HFIAS: as the number of months with adequate food provisioning increases from 0 to 12, the HFIAS score, indicative of food insecurity, decreases progressively. The smoothed curve, supported by the 95% CI, underscores a robust linear correlation between these variables and reinforces the reliability of the HFIAS score as a proxy for year‐round food security, effectively capturing variations in household food access across the observed timeframe.

### 3.4 Women's Dietary Diversity

**FIGURE 3:**
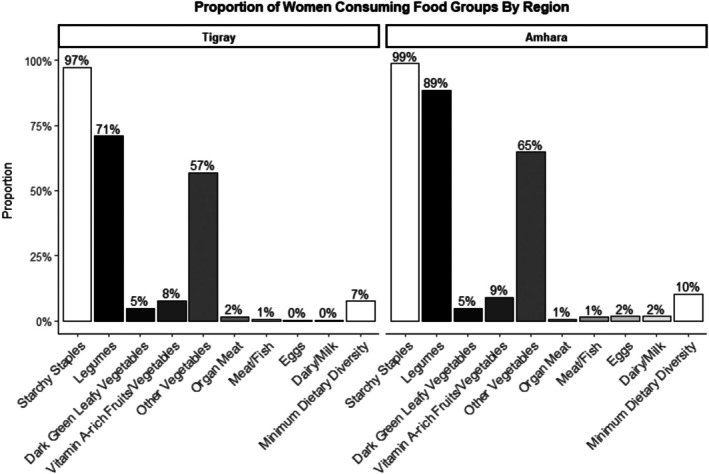
Proportion of pregnant and lactating women who reported consumption of specific food groups in the previous 24 h by regions.

Figure 3 illustrates the proportion of women consuming various food groups by region among pregnant and lactating women (PLW) in Ethiopia. In Tigray, 97% of women consumed starchy staples, while 71% ate legumes and pulses in the 24‐h preceding their report. The consumption of dark green leafy vegetables was just 5%, and only 8% consumed vitamin A‐rich fruits and vegetables. Additionally, 57% of women included other vegetables in their diet, but nearly no women consumed animal‐source foods; only 7% met the minimum dietary diversity standards. A similar pattern was observed in Amhara, with 99% of the population consuming staple grains and 89% eating legumes and pulses. Dark green leafy vegetable consumption remained low at 5%, and only 9% of the population consumed vitamin A‐rich fruits and vegetables. Furthermore, 65% of participants included other vegetables in their diet, while the consumption of animal‐source foods was limited to less than 2%; only 10% of participants met the minimum dietary diversity criteria.

**FIGURE 4:**
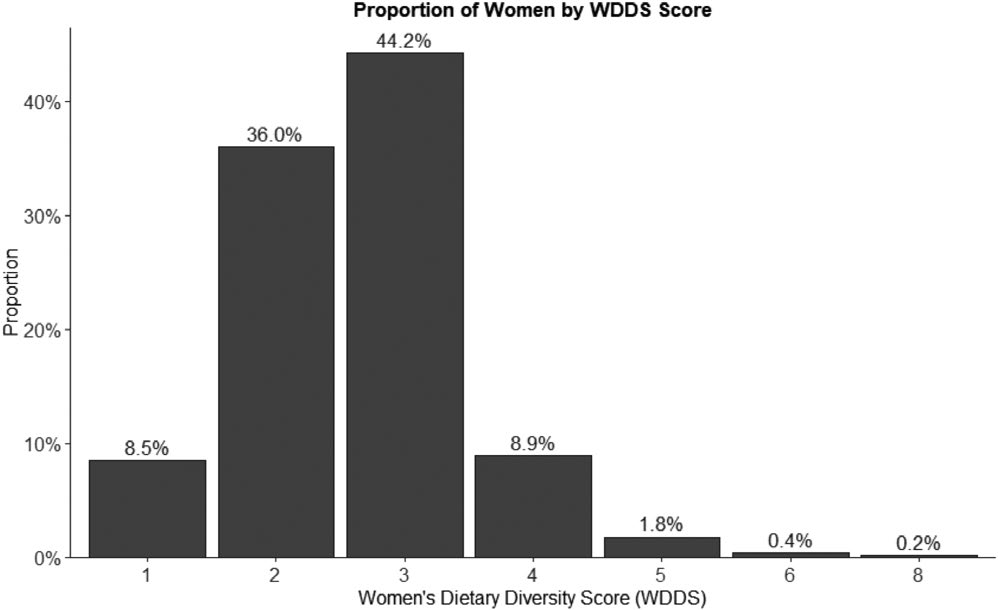
Proportion of pregnant and lactating women consuming different scores of the Women's Dietary Diversity Score (WDDS).

Figure 4 illustrates the distribution of women based on their WDDS among PLW. The percentage of women with a WDDS of 1 was 8.5%, increasing to 36.0% for a score of 2 and peaking at 44.2% for a score of 3. After that, the percentage decreased to 8.9% for a score of 4, 1.8% for a score of 5, 0.4% for a score of 6, and 0.2% for a score of 8.

**FIGURE 5:**
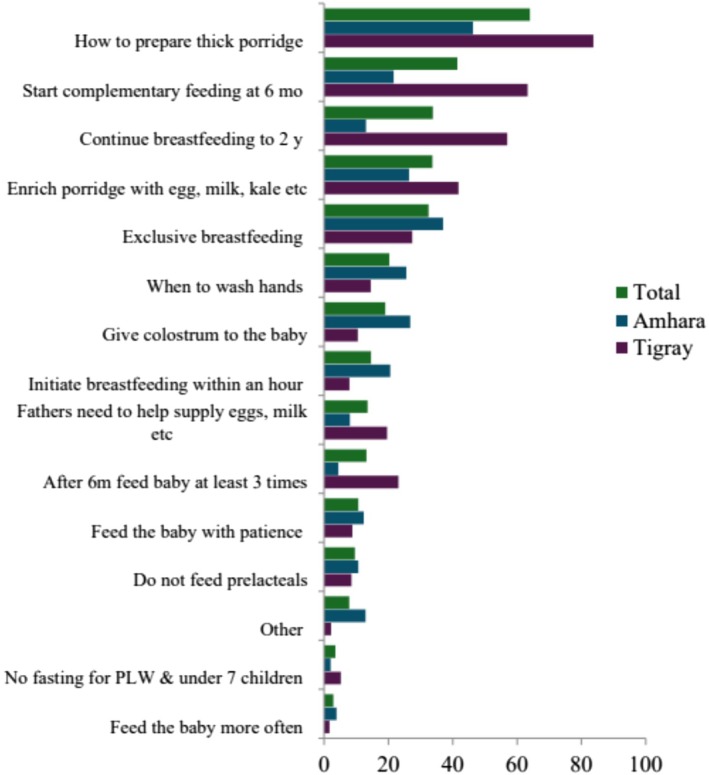
Topics of nutrition messages that were discussed during the community conversation between PLW and health development agents over the previous 3 months.

Figure 5 illustrates the percentage of pregnant and lactating women who discussed a specific maternal and young child feeding message, designed as part of social and behavioral change communication material, delivered to the community by health development agents. The highest awareness across all groups is for preparing thick porridge (around 80% total, 70% among Amhara, and 90% among Tigray) and starting complementary feeding at 6 months (70% total, 60% among Amhara, and 80% among Tigray). Practices such as frequent feeding after 6 months and avoiding pre‐lacteals demonstrate moderate awareness (20%–40%), while knowledge of not fasting for pregnant or lactating women and children under 7 is notably low (below 10% across all groups). Tigray generally exhibits higher awareness than Amhara for most practices.

### 3.5 Production Diversity

Table 4 presents descriptive data on the proportion of households producing various food groups by region. Cultivated crops were categorized into various food groups and tabulated by households that produced them. The results show that almost all households cultivated at least one crop in the cereals food group (99% in Tigray and 98% in Amhara), followed by legumes (19.4% in Tigray and 56.2% in Amhara). The Pearson correlation test reveals significant variation between the cultivation of legumes in the Amhara and Tigray regions (*p* < 0.001). Both regions have a small number of households engaged in fruit and vegetable production, as presented in Table 4.

**TABLE 4:** Types of crops cultivated by households in Tigray and Amhara regions (*N* = 2036).

Variables (*N* = 2036)	Tigray (*N* = 1108)		Amhara (*N* = 928)		Pearson's *χ* ^2^
	*n*	%	*n*	%	*p*
Food groups cultivated					
Cereals	1101	99.4	911	98.2	0.0396
Legumes and nuts	215	19.4	521	56.2	< 0.001
Seeds/oil crops	89	8.0	62	6.6	0.4651
Root and tuber crops	4	0.3	63	6.7	< 0.001
Vegetables	78	7.0	61	6.6	0.8842
Fruits	14	1.2	8	0.87	0.5892
Cash crops	20	1.8	73	7.8	< 0.001

### 3.6 Risk Factors of Food Insecurity in the Seqota Declaration Areas

Table 5 presents the risk factors for food insecurity (moderate or severe) among 2036 households in the Seqota Declaration Innovative Phase Survey Areas of Ethiopia, utilizing logistic regression models. The bivariate analysis revealed several factors significantly or marginally associated with food insecurity. Households in Amhara showed marginally higher odds of food insecurity than those in Tigray (OR 1.20, 95% CI 0.99–1.45, *p* = 0.058). Female‐headed households had higher odds of experiencing food insecurity than male‐headed households (OR 1.51, 95% CI 1.15–2.10, *p* = 0.003). Muslim household heads experienced marginally lower odds of food insecurity compared to Orthodox household heads (OR 0.52, 95% CI 0.26–1.04, *p* = 0.066). Older household heads faced a greater risk, with those aged 41–64 years (OR 1.47, 95% CI 1.19–1.81, *p* < 0.001) and those over 65 years (OR 1.59, 95% CI 1.20–2.10, *p* = 0.001) having higher odds compared to those aged 15–40 years. Wealth quintiles exhibited a clear gradient in terms of food insecurity. The poorest group (Q1) had the highest odds of experiencing food insecurity, with an odds ratio of 2.16 (95% CI 1.54–3.03; *p* < 0.001). This was followed by the second quintile (Q2), which had an odds ratio of 1.81 (95% CI 1.29–2.55; *p* = 0.001), and the third quintile (Q3), with an odds ratio of 1.66 (95% CI 1.17–2.37; *p* = 0.005). In contrast, the wealthiest group (Q5) had the lowest odds of food insecurity, as expected.

**TABLE 5:** Risk factors for moderate or severe food insecurity among households in the Seqota Declaration innovative phase survey areas, Ethiopia (*N* = 2036): Bivariate and multivariable logistic regression results.

Characteristic	Food insecure	Bivariate logistic regression		Multivariable logistic regression	
	%	OR [95% CI]	*p*	AOR [95% CI]	*p*
Region					
Tigray	55.0	1		1	
Amhara	59.5	1.20 [0.99–1.45]	0.058	1.34 [0.97–1.85]	0.071
Gender of household head					
Male	55.5	1		1	
Female	65.4	1.51 [1.15–2.10]	**0.003**	1.00 [0.71–1.41]	0.979
Religion of household head					
Orthodox	57.4	1		1	
Muslim	41.4	0.52 [0.26–1.04]	0.066	0.60 [0.34–1.06]	0.076
Age of household head					
15–40 years	51.0	1		1	
41–64 years	60.4	1.47 [1.19–1.81]	**< 0.001**	1.85 [1.47–2.34]	**< 0.001**
Above 65 years	62.4	1.59 [1.20–2.10]	**0.001**	1.70 [1.23–2.34]	**0.001**
Wealth quintiles					
Q1 (poorest)	64.6	2.16 [1.54–3.03]	**< 0.001**	2.33 [1.59–3.42]	**< 0.001**
Q2	60.6	1.81 [1.29–2.55]	**0.001**	1.75 [1.21–2.51]	**0.003**
Q3	58.5	1.66 [1.17–2.37]	**0.005**	1.62 [1.05–2.50]	**0.030**
Q4	45.4	0.98 [0.69–1.40]	0.915	0.97 [0.67–1.45]	0.926
Q5 (Richest)	45.8	1			
Household head education					
No formal education	60.2	2.04 [1.16–3.58]	**0.013**	1.09 [0.50–2.37]	0.822
Primary and above	49.5	1.32 [0.74–2.36]	0.351	0.99 [0.45–2.16]	0.983
Above high school	42.6	1		1	
Cash or food assistance received in the past year					
No	54.8	1		1	
Yes	60.4	1.25 [1.03–1.53]	**0.028**	0.96 [0.74–1.26]	0.788
Size of arable land (hectares)		0.85 [0.74–0.96]	**0.010**	0.91 [0.82–1.01]	0.077
Tropical Livestock Unit (TLU)		0.86 [0.81–0.91]	**< 0.001**	0.86 [0.80–0.93]	**< 0.001**
Improved land and water management practices					
No	66.8	1		1	
Yes	54.6	0.60 [0.47–0.76]	**< 0.001**	0.73[0.54–1.00]	**0.048**
Small‐scale irrigation					
No	58.6	1		1	
Yes	46.8	0.62 [0.46–0.83]	**0.001**	0.74 [0.50–1.11]	0.127
Use of improved seeds/seedlings					
No	57.4	1.27 [0.84–1.91]	0.256		
Yes	51.5	1			
Use of fertilizers					
No	67.1	1.61 [1.17–2.22]	**0.004**	1.18 [0.85–1.64]	0.306
Yes	55.9	1		1	
Food groups cultivated (count)		0.75 [0.66–0.86]	**< 0.001**	0.83 [0.68–0.99]	**0.046**

*Note:* Bivariate analysis uses unadjusted logistic regression. Multivariable analysis adjusts for all listed covariates. Bolded *p*‐values indicate statistical significance (*p* < 0.05).

Abbreviations: AOR, adjusted odds ratio; CI, confidence interval; OR, odds ratio.

Households with a household head who had no formal education had higher odds of experiencing food insecurity (OR 2.04, 95% CI 1.16–3.58, *p* = 0.013) compared to those with an education above high school. Receiving cash or food assistance was associated with a higher risk of experiencing food insecurity (OR 1.25, 95% CI 1.03–1.53, *p* = 0.028). Larger arable land size (OR 0.85, 95% CI 0.74–0.96, *p* = 0.010) and higher Tropical Livestock Unit (TLU) (OR 0.86, 95% CI 0.81–0.91, *p* < 0.001) were associated with lower odds of food insecurity. Households applying improved land and water management practices had lower odds (OR 0.60, 95% CI 0.47–0.76, *p* < 0.001), as did small‐scale irrigation beneficiaries (OR 0.62, 95% CI 0.46–0.83, *p* = 0.001). Not using fertilizers increased the odds of food insecurity (OR 1.61, 95% CI 1.17–2.22, *p* = 0.004), while a higher count of food groups cultivated reduced the odds (OR 0.75, 95% CI 0.66–0.86, *p* < 0.001).

**FIGURE 6:**
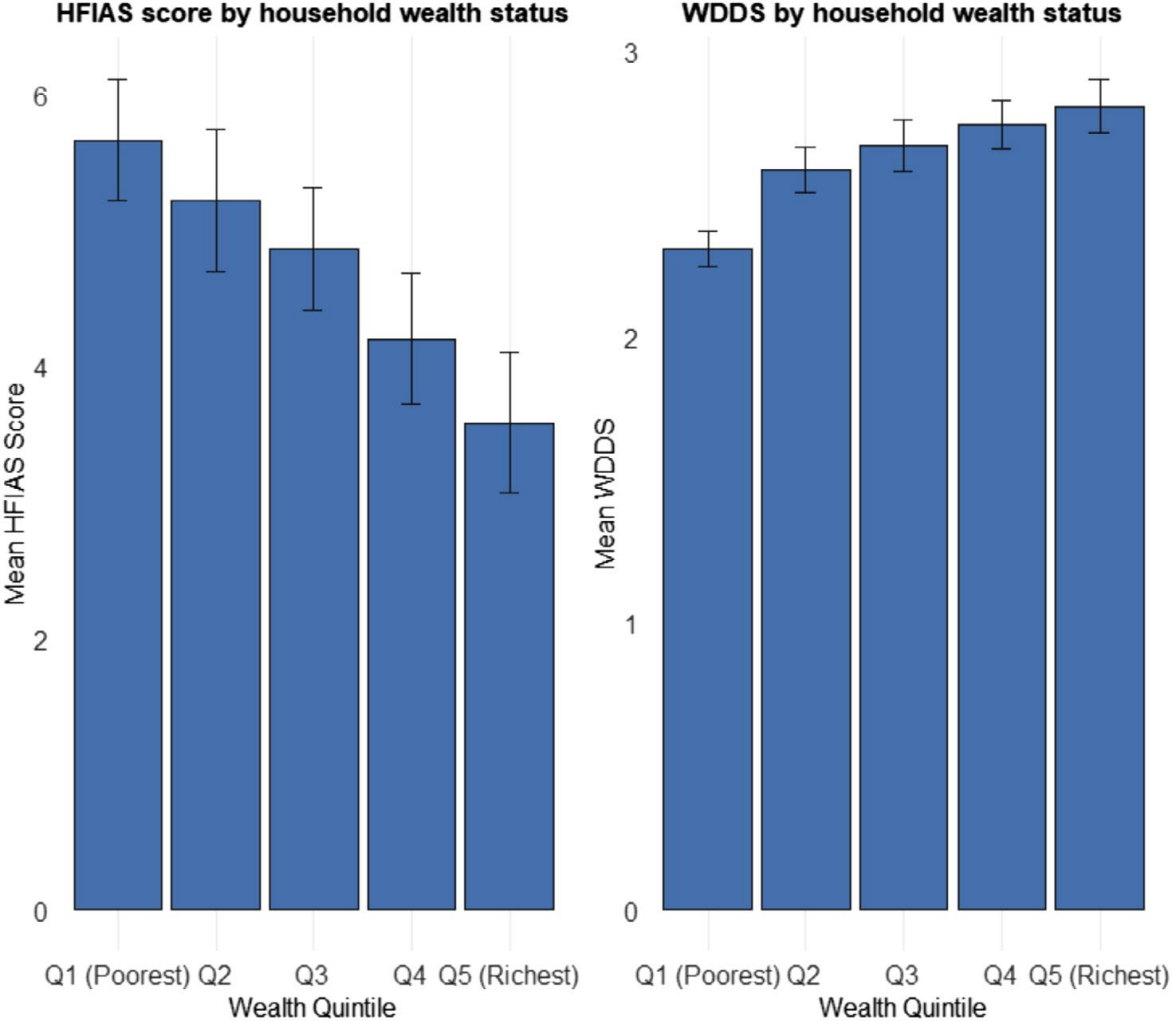
The mean household food insecurity access score (HFIAS) and women's dietary diversity score (WDDS), along with standard error bars, are presented by wealth quintile categories for pregnant and lactating women in Ethiopia (*N* = 2036). The left panel illustrates the mean HFIAS score, while the right panel depicts the mean WDDS, both organized by wealth quintiles, ranging from Q1 (poorest) to Q5 (richest).

Figure 6 illustrates the average HFIAS score and Women's Dietary Diversity Score (WDDS) with standard error bars across wealth quintiles among pregnant and lactating women. The average HFIAS score decreased from 5.7 in Q1 (Poorest) to 3.6 in Q5 (Richest). This consistent decline in HFIAS scores indicates that households with greater asset ownership experienced significantly lower instances of food insecurity in the past 30 days compared to those with lower wealth status. In contrast, the average WDDS increased from 2.3 in Q1 (Poorest) to 3.0 in Q5 (Richest).

Several factors maintained their statistical significance in the multivariable logistic model, while the significance of some variables was only observed in the bivariate model (Tables 5 and 6). For instance, household heads aged 41–64 years had higher odds of experiencing food insecurity (AOR 1.85, 95% CI 1.47–2.34, *p* < 0.001), as did those over the age of 65 (AOR 1.70, 95% CI 1.23–2.34, *p* = 0.001), compared to individuals aged 15 to 40 years. The wealth quintiles were also significant in both the bivariate and multivariable models. The poorest quintile (Q1) had the highest odds (AOR 2.33, 95% CI 1.59–3.42, *p* < 0.001), followed by the second quintile (Q2) (AOR 1.75, 95% CI 1.21–2.51, *p* = 0.003) and the third quintile (Q3) (AOR 1.62, 95% CI 1.05–2.50, *p* = 0.030), when compared to the wealthiest quintile (Q5). Additionally, a higher Tropical Livestock Unit (TLU) count was associated with lower odds of food insecurity (AOR 0.86, 95% CI 0.80–0.93, *p* < 0.001). Households that utilized improved land and water management practices demonstrated lower odds of food insecurity (AOR 0.73, 95% CI 0.54–1.00, *p* = 0.048). Additionally, a greater diversity in the number of food groups cultivated was linked to reduced odds (AOR 0.83, 95% CI 0.68–0.99, *p* = 0.046).

**TABLE 6:** Factors associated with minimum dietary diversity (MDD‐W) and women's dietary diversity score (WDDS) in Seqota Declaration survey areas, Ethiopia (*N* = 642): Bivariate and multivariable Poisson regression results.

Characteristic	% Achieving MDD‐W	Bivariate Poisson regression: WDDS		Multivariable Poisson regression: WDDS	
		IRR [95% CI]	*p*	AIRR [95% CI]	*p*
Region					
Tigray	6.9	1		1	
Amhara	10.2	1.13 [1.05–1.22]	0.001	1.06 [0.94–1.19]	0.292
Gender of household head					
Male	8.4	1			
Female	18.6	1.06 [0.93–1.21]	0.372		
Religion of household head					
Orthodox	8.5	1			
Muslim	20.7	1.05 [0.89–1.24]	0.88543		
Wealth quintiles					
Q1 (poorest)	4.1	1		1	
Q2	7.5	1.11 [1.01–1.22]	0.002	1.10 [0.94–1.27]	0.227
Q3	10.8	1.15 [1.03–1.29]	0.011	1.14 [0.97–1.32]	0.103
Q4	13.1	1.19 [1.07–1.31]	0.001	1.17 [1.00–1.37]	0.044
Q5 (richest)	11.9	1.21 [1.10–1.34]	< 0.001	1.19 [1.01–1.40]	0.043
PLW education					
No formal education	8.0	1			
Primary and above	10.2	1.05 [0.99–1.13]	0.101		
Cash or food assistance received					
No	10.0	1		1	
Yes	6.9	0.94 [0.88–1.00]	0.046	0.94 [0.87–1.09]	0.307
Exposure to MIYCN messages					
No	6.6	1		1	
Yes	11.1	1.08 [1.01–1.15]	0.027	1.05 [0.94–1.17]	0.365
Food security status (HFIAS)					
Secure (secure/mild)	10.2	1		1	
Insecure (moderate/severe)	7.6	0.91 [0.86–0.97]	0.005	0.98 [0.87–1.09]	0.729
Number of months of reported adequate household food provisioning (MAHFP)					
< 4 months	5.3	1		1	
5–8 months	4.5	0.99 [0.87–1.13]	0.934	0.96 [0.74–1.25]	0.757
9–12 months	12.1	1.14 [1.01–0.97]	0.029	1.06 [0.82–1.38]	0.637
Size of arable land (hectares)		1.05 [1.04–1.06]	< 0.001	1.04 [1.01–1.07]	0.017
Total Livestock Unit (TLU)		1.01 [0.99–1.02]	0.296		
Improved land and water management practice					
No	11.0	1			
Yes	8.3	1.06 [0.97–1.16]	0.177		
Small‐scale irrigation beneficiary					
No	8.8	1			
Yes	9.1	1.00 [0.92–1.09]	0.919		
Use of improved seeds/seedlings					
No	6.1	1.10 [0.89–1.37]	0.358		
Yes	9.0	1			
Use of fertilizers					
No	4.5	1		1	
Yes	9.4	1.14 [1.05–1.24]	0.003	1.11 [0.93–1.32]	0.224
Food groups cultivated (count)		1.09 [1.05–1.13]	< 0.001	1.05 [0.97–1.13]	0.178

### 3.7 Factors Influencing Women's Dietary Diversity in the Seqota Declaration Areas

Table 6 presents factors influencing Women's Dietary Diversity Score (WDDS) and Minimum Dietary Diversity for Women (MDD‐W) among 642 women in the Seqota Declaration Survey Areas of Ethiopia, using Poisson regression models.

The bivariate analysis revealed several significant associations with women's dietary diversity. Regional differences were apparent, with women in Amhara showing a higher likelihood of achieving WDDS than those in Tigray (IRR 1.13; 95% CI 1.05–1.22; *p* = 0.001). A clear wealth gradient emerged, as women from wealthier households consistently displayed higher IRRs for WDDS; the wealthiest quintile (Q5) showed the most significant increase compared to the poorest (Q1) (IRR 1.21, 95% CI 1.10–1.34, *p* < 0.001). Interestingly, receiving cash or food assistance was marginally associated with a slight decrease in WDDS (IRR 0.94; 95% CI 0.88–1.00; *p* = 0.046). Positive influences included exposure to Maternal, Infant, and Young Child Nutrition (MIYCN) messages (IRR 1.08, 95% CI 1.01–1.15, *p* = 0.027) and improved food security, with households experiencing moderate to severe food insecurity having lower dietary diversity (IRR 0.91, 95% CI 0.86–0.97, *p* = 0.005). Conversely, households with 9–12 months of adequate food provisioning showed notably higher MDD‐W (IRR 1.14; 95% CI 1.01–1.28; *p* = 0.029) than those with fewer than 4 months of adequate food provisioning. Agricultural factors also played a strong role: larger arable land sizes (IRR 1.05, 95% CI 1.04–1.06, *p* < 0.001), fertilizer use (IRR 1.14, 95% CI 1.05–1.24, *p* = 0.003), and cultivating a higher number of food groups (IRR 1.09, 95% CI 1.05–1.13, *p* < 0.001) were all positively linked to higher MDD‐W.

### 3.8 Key Predictors in Multivariable Analysis

When considering these factors simultaneously in the multivariable Poisson regression model, a more refined picture emerged, with fewer yet crucial predictors remaining significant. The strong influence of wealth persisted, as only the two wealthiest quintiles, Q4 (adjusted IRR [AIRR] 1.17, 95% CI 1.00–1.37, *p* = 0.044) and Q5 (AIRR 1.19, 95% CI 1.01–1.40, *p* = 0.043), continued to show significantly higher adjusted IRRs for MDD‐W compared to the poorest (Q1). Additionally, a larger arable land size remained a significant positive predictor (AIRR 1.04, 95% CI 1.01–1.07, *p* = 0.017), underscoring the enduring importance of agricultural resources for women's dietary diversity in these households.

**FIGURE 7:**
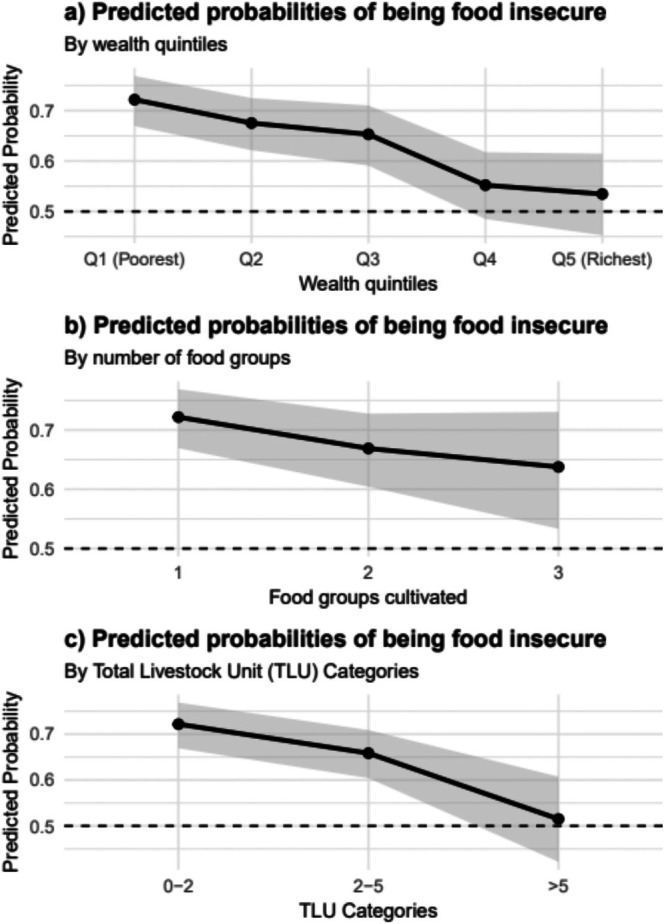
Predicted probabilities of being food insecure by socioeconomic and agricultural factors. (a) Predicted probabilities by wealth quintiles. (b) Predicted probabilities by the number of food groups cultivated, with values at 1, 2, and 3 groups. (c) Predicted probabilities by total livestock unit (TLU) categories. Shaded areas represent 95% confidence intervals, estimated from a survey‐weighted model.

Figure 7 clearly illustrates the inverse relationship between key socioeconomic and agricultural indicators and the predicted probability of household food insecurity. As socioeconomic status (SES) rises, the likelihood of food insecurity steadily decreases, with the lowest SES quintile facing a 70% probability compared to 53% for the highest (Figure 7a). Similarly, a higher number of food groups cultivated by households is associated with a lower probability of food insecurity, decreasing from 72% for those consuming just one group to 63% for those consuming three (Figure 7b). Furthermore, increased livestock ownership, measured by Tropical Livestock Units (TLU), serves as a strong protective factor, with the probability of food insecurity decreasing from 71% in the 0–2 TLU category to 51% for households with over 5 TLU (Figure 7c). Collectively, these panels emphasize that higher socioeconomic standing, greater dietary or production diversity, and increased livestock assets are consistently linked to a significantly reduced probability of food insecurity.

**FIGURE 8:**
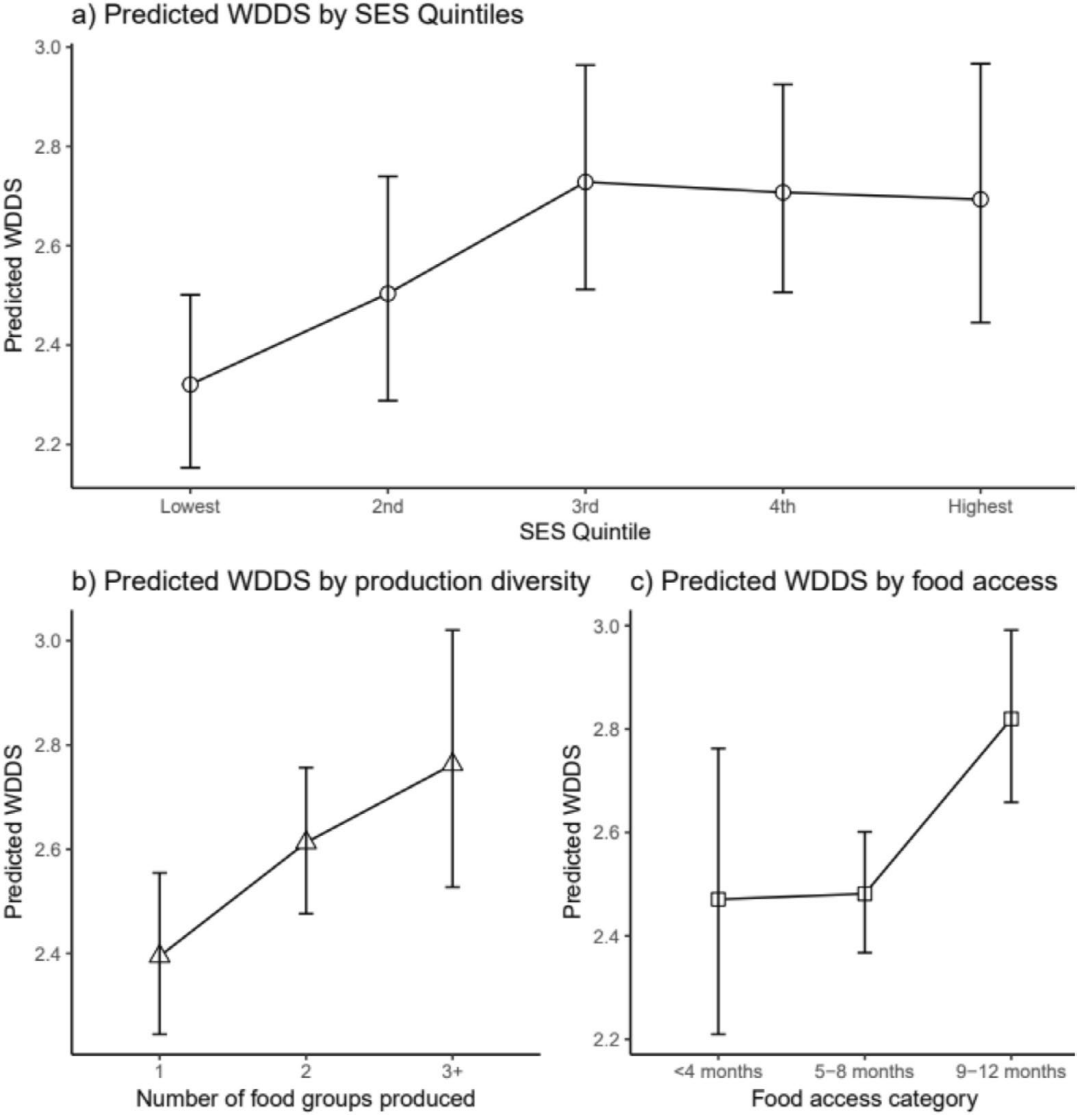
Predicted Women's Dietary Diversity Score (WDDS) by socioeconomic and agricultural factors. (a) Predicted WDDS across quintiles of socioeconomic status (SES). (b) Predicted WDDS based on the variety of food groups produced, reflecting production diversity. (c) Predicted WDDS segmented by food access category, indicating the reported number of months with adequate household food access. Estimates are derived from a survey‐weighted model, with error bars illustrating confidence intervals.

Figure 8 illustrates how socioeconomic and agricultural factors influence the predicted Women's Dietary Diversity Score (WDDS). A higher socioeconomic status (SES) is positively associated with WDDS, exhibiting a clear upward trend from the lowest quintile (approximately 2) to the third quintile (approximately 3), after which it stabilizes or slightly declines in the highest quintiles (Figure 8a).

Increased production diversity, as indicated by the number of food groups produced, correlates with a higher WDDS, increasing from two for those who produce one food group to around three for those who produce three or more food groups (Figure 8b). Additionally, better food access, as indicated by longer durations of food availability, is associated with a higher Weighted Dietary Diversity Score (WDDS). Households with 9–12 months of food access exhibit a noticeably higher WDDS compared to those with < 4 months (Figure 8c). Together, these panels illustrate that socioeconomic well‐being, diversified agricultural production, and consistent food access are essential drivers of improved dietary diversity among women.

## 4 Discussion

The findings of this study reveal the interaction of socio‐demographic, economic, and agricultural factors influencing household food insecurity and women's dietary diversity in the SD areas of Tigray and Amhara, regions known for their infertile land and chronic food shortages.

### 4.1 Household Food Insecurity: Prevalence and Risk Factors

The study revealed a significant prevalence of food insecurity in both the Tigray and Amhara regions across the pilot woredas of the SD program (see Table 5). Using the Household Food Insecurity Access Scale (HFIAS), 35.4% of households in Tigray and 37.8% in Amhara experienced moderate food insecurity, while severe food insecurity affected 19.6% and 21.7% of households, respectively (Table 3). These figures align with previous research in Ethiopia, where food insecurity has long been recognized as a critical problem (Sani and Kemaw 2019; Sisha 2020; Telila and Sima 2024). For instance, a nationwide study by Telila and Sima (2024) reported a comparable but slightly lower prevalence of moderate to severe food insecurity (52%) in Ethiopia. The high prevalence of food insecurity in the study areas may be attributed to limited agricultural productivity and recurrent droughts that characterize these areas (The Federal Democratic Republic of Ethiopia Seqota Declaration 2016). The Months of Adequate Household Food Provisioning (MAHFP) metric revealed significant regional differences (*p* < 0.001); however, Amhara showed a higher prevalence of mild food insecurity (35.5%) compared to Tigray (22.3%), while Tigray had more severe cases (13.9% vs. 8.8%) (Table 3). This discrepancy suggests that while Amhara households may experience more frequent but less severe food shortages, Tigray households face more acute and severe episodes, possibly due to differences in agroecological conditions or access to safety nets.

The bivariate analysis revealed several significant factors associated with food insecurity among households, encompassing demographic, socioeconomic, and agricultural dimensions (Table 5). Notably, female‐headed households had 51% higher odds of experiencing food insecurity (OR = 1.51, *p* = 0.003) than their male counterparts. The higher risk among women may reflect the broader structural disadvantages faced by women in Ethiopia, including limited access to social assets, economic opportunities, and income‐generating activities (Negesse et al. 2020). In many rural areas, cultural norms dictate that family assets, particularly agricultural land, are inherited by male descendants. This gendered disparity in resource ownership and economic engagement contributes to heightened vulnerability among female‐headed households.

The findings suggest that older household heads are at a higher risk of experiencing food insecurity compared to younger ones. This may be attributed to the labor‐intensive nature of livelihoods in rural communities, where older individuals may have reduced capacity to participate in diverse income‐generating activities, thereby limiting their ability to adequately provide for their households. This finding aligns with previous studies that link increasing age with a higher risk of food insecurity (Gezimu Gebre 2012; Mengistu et al. 2021).

The study reveals a notable disparity in food insecurity across various socioeconomic variables. Household wealth status is an important predictor, with the poorest quintile (Q1) having more than twice the odds of experiencing insecurity (OR 2.16, *p* < 0.001) compared to the wealthiest quintile (Q5). The odds of being food insecure progressively decrease with increasing wealth. A lack of formal education of the household head doubled the odds (OR 2.04, *p* = 0.013) of being food insecure. This finding aligns with the findings of other studies conducted in similar settings, which have been linked to better food security (Gezimu Gebre 2012; Mengistu et al. 2021; Sisha 2020; Tamiru et al. 2016). To address this, targeted educational interventions, such as adult literacy programs or agricultural training tailored to local farming practices, could empower household heads in Ethiopia to enhance income generation and access to nutrient‐adequate diets. Receipt of cash or food assistance was associated with 25% higher odds of experiencing food insecurity (OR 1.25, *p* = 0.028), suggesting that effective targeting can serve as a safety net for households already facing challenges. Safety net assistance primarily provides a small amount of cash assistance and staple foods (Welteji et al. 2017) with limited impact on food security and dietary diversity (Feyisa 2021). The correlation between food insecurity and safety net beneficiary status may stem from the Household Food Insecurity Access Scale (HFIAS), which measures perceived food insecurity, potentially explaining why assistance recipients report elevated concerns about food security.

The relationship between agricultural practices and food security outcomes was a primary focus of this study. The multivariable logistic regression model included several agricultural variables, such as the size of arable land owned, livestock holdings (measured in Tropical Livestock Units, or TLU), the adoption of at least one improved land or water management practice (e.g., tree or shrub planting, terracing, drainage systems, soil or stone bunding, or gully treatment), access to small‐scale irrigation, fertilizer use, and the diversity of food groups cultivated by each household.

Several variables retained statistical significance in both bivariate and multivariable models. These factors include household wealth quintile, with the poorest households (Q1) facing more than twice the odds of food insecurity compared to the wealthiest households (OR 2.34, 95% CI 1.78–3.09, *p* < 0.001). This finding highlights the role of economic disparities in shaping access to nutrient‐adequate diets within Ethiopia's food environment, suggesting that targeted interventions, such as income‐support programs or subsidies for nutrient‐rich foods, could help mitigate food insecurity among low‐income households. Figure 6 further supports this pattern, showing a steady decline in mean Household Food Insecurity Access Scale (HFIAS) scores from 5.7 in the lowest wealth quintile (Q1) to 3.6 in the highest (Q5), illustrating how asset ownership mitigates food insecurity. This result aligns with global evidence that links poverty to food insecurity (FAO et al. 2024). The predicted probability estimate, illustrated in Figure 7a, reveals a decrease in the probability of food insecurity based on wealth status, dropping from 70% in the poorest households to 53% in the wealthiest households. These findings align with initial expectations and are consistent with prior studies. For instance, Gizaw et al. (2025) stated that higher wealth status enhances access to agricultural inputs and diversified income sources, thereby reducing vulnerability to food insecurity in Ethiopian rural households. The authors argue that wealthier households are better positioned to invest in productivity‐enhancing technologies, such as improved seeds and irrigation, which stabilize food production and access. Similarly, household ownership of productive assets, including livestock and farm equipment, serves as a buffer against economic shocks (Shifat et al. 2025), thereby explaining the protective effect of wealth against food insecurity. In contrast, poorer households, constrained by limited resources, face a higher risk of food shortages, particularly during lean seasons, as noted by Hadley et al. (2011). These studies collectively underscore the critical role of economic status in shaping food security outcomes, reinforcing the observed wealth gradient in this analysis.

The findings of this study highlight the crucial role of diversity in agricultural production and livestock in mitigating food insecurity. Higher Tropical Livestock Units (TLU) (AOR 0.86, 95% CI 0.80–0.93, *p* < 0.001) and increased production diversity (AOR 0.83, 95% CI 0.68–0.99, *p* = 0.046) are associated with a lower likelihood of food insecurity (see Table 5). Livestock are a crucial source of income, providing resilience during crises and allowing smallholder farmers to improve their agricultural activities. For instance, previous research has shown that oxen ownership significantly enhances food production and food security for households (Bedeke 2012). Figure 7c illustrates a considerable reduction in the likelihood of food insecurity from 71% for households with 0–2 TLU to 51% for those with over 5 TLU, emphasizing the importance of livestock as a buffer against food shortages. This finding is supported by Megersa et al. (2014), who indicated that livestock ownership strengthens household resilience in Ethiopia. Several other studies suggest that livestock ownership is crucial for enhancing household food security (Asenso‐Okyere et al. 2013; Sani and Kemaw 2019; Tamiru et al. 2016). Additionally, the protective effect of production diversity (Figure 7b) signifies that diversifying crops can stabilize food access, reinforcing the claims made by Jones et al. (2014), who argue that production diversity is a key strategy for enhancing food security in smallholder systems. A likely explanation for this observation is that cultivating fewer crops may restrict the household's options in the event of crop failure, low yields, or other potential disasters, compared to growing a greater variety of crops, which may offer more alternatives during such shocks.

Land and water management practices are linked to lower odds of food insecurity compared to those who do not engage in these practices. Studies have shown that poor soil management is associated with low crop yields and food insecurity (Sani and Kemaw 2019; Sisha 2020). Given the infertile and rocky terrain of the Tekeze River Basin study areas (The Federal Democratic Republic of Ethiopia 2016), the Seqota Declaration initiative should promote sustainable community practices by supporting traditional land and water management techniques while integrating innovative conservation methods. Current practices, such as stone bunding and terracing, are highly labor‐intensive, necessitating the adoption of efficient, scalable approaches to enhance agricultural resilience and reduce labor burdens.

### 4.2 Women's Dietary Diversity: Patterns and Determinants

Women's dietary diversity among PLW was notably low, with only 7% in Tigray and 10% in Amhara meeting minimum dietary diversity standards (Figure 3). Most of such women consumed at least one food item in the starchy staple food group (97% in Tigray, 99% in Amhara) and legumes (71% in Tigray, 89% in Amhara) but the intake of nutrient‐rich foods such as dark green leafy vegetables (5% in both regions) and animal‐source foods (< 2%) remained minimal. This pattern reflects broader dietary challenges in Ethiopia, where reliance on staples predominates due to limited access to diverse and often more expensive foods (Sibhatu and Qaim 2018). Figure 4 shows that 44.2% of PLW had a WDDS of 3, with only 0.2% reaching a score of 8, indicating a significant nutritional gap for this vulnerable group, which can have adverse implications for maternal and child health outcomes (Black et al. 2013). The Food and Agriculture Organization recommends daily consumption of foods from at least five food groups (Food and Agriculture Organization of the United Nations (FAO) 2016).

Multivariable Poisson regression (Table 6) identified wealth and arable land size as key predictors of WDDS. Women in the wealthiest quintile exhibited higher dietary diversity compared to those in the poorest quintile (Q1), a trend visually confirmed by Figure 8a, which shows WDDS increasing from approximately two food groups in the lowest quintile to three food groups in the third quintile, stabilizing through the fourth and fifth quintiles. Similar to the trend in food insecurity, this study also identified household wealth as a strong determinant of women's dietary diversity, highlighting the critical role of assets in enhancing household food security and women's dietary diversity. The wealth index was derived from various household assets, including ownership of livestock, land, and other valuable items, and serves as a proxy for overall economic status. Households in higher wealth quintiles may have a greater capacity to purchase or produce food compared to those in lower quintiles. Productive assets—such as livestock, agricultural equipment, and other essential items—were also significantly associated with a lower risk of food insecurity (Table 5).

A larger area of arable land (AIRR 1.04, 95% CI 1.01–1.07, *p* = 0.017) had a positive influence on WDDS, likely by enabling greater production diversity (Figure 8b). Women's dietary diversity increased with a higher number of food groups cultivated by the household. These findings align with those of Sibhatu and Qaim (2017), who argue that access to both land and economic resources is essential for enhancing dietary diversity in rural settings.

Interestingly, although exposure to dietary messaging, including community gatherings regarding Maternal, Infant, and Young Child Nutrition (MIYCN) by health development agents, was significant in the bivariate analysis with WDDS, it did not remain significant in the multivariable model (Table 6). This indicates that while nutrition education may raise awareness, structural barriers such as poverty and limited food access can lessen its effectiveness (Ruel et al. 2018). The assessment of nutrition messages received by participants revealed that most focused exclusively on child nutrition, with only a few mothers reporting messages about avoiding fasting during pregnancy and lactation (Figure 5). This finding highlights the need to expand social and behavior change communication (SBCC) efforts to include messages tailored specifically to women's nutritional needs. Incorporating such targeted messaging could enhance both maternal and child nutrition outcomes, supporting the goals of Ethiopia's Seqota Declaration to address nutritional vulnerability.

Production diversity was limited, with almost all households cultivating cereals (99.4% in Tigray and 98.2% in Amhara); significantly fewer households grew legumes (19.4% in Tigray and 56.2% in Amhara) or vegetables (7.0% in Tigray and 6.6% in Amhara) (Table 4). The notable regional difference in legume cultivation (*p* < 0.001) may reflect agroecological variations, as the Amhara region has the highest legume production in Ethiopia. This is possibly due to a suitable climate for legumes, as noted by Neda (2020). However, the low involvement in fruit and vegetable production in both regions restricts dietary diversity, especially for nutrient‐dense foods, which supports the findings by Sibhatu et al. (2015) that Ethiopian smallholders prioritize staple crops over diverse production.

Overall, significant bivariate associations were initially observed between larger land sizes and the practice of small‐scale irrigation, with each factor reducing the risk of being food insecure. However, multivariable analysis revealed no significant statistical relationship for land size or irrigation practices. A possible explanation is that the small average landholding of 2.23 ha limits economies of scale in agricultural production (Gizaw et al. 2025), thereby reducing their impact on food security outcomes. Gizaw et al. (2025) further argue that the limited success of small‐scale irrigation schemes—often attributed to inadequate infrastructure and poor water management—diminishes their influence on food security, highlighting the necessity for improved irrigation strategies. In contrast, land size demonstrated a modest positive correlation with women's dietary diversity score, suggesting that larger landholdings may enhance dietary diversity through diversified crop production. This study's findings highlight the need to address barriers to food and nutrition security in Ethiopia by promoting crop diversification, improving irrigation efficiency, and fostering income diversification. These strategies can enhance rural livelihoods, strengthen resilience in shocks, and improve access to nutrient‐adequate diets.

### 4.3 Implications

These findings have important implications for designing effective interventions to improve food security and dietary diversity in Ethiopia, especially in remote, resource‐scarce areas such as the Tekeze River Basin woredas targeted by the Seqota Declaration pilot interventions.

The strong association between wealth, livestock ownership, production diversity, and reduced food insecurity suggests that interventions should prioritize asset‐building strategies, such as supporting smallholder farmers with improved livestock varieties or support for crop diversification, to address food insecurity. The positive correlation between land size, production diversity, and dietary diversity underscores the importance of equitable land access policies in enhancing food production and nutrition outcomes. However, the limited impact of MIYCN messages suggests that nutrition education should be designed to target both women and children; yet, education alone is insufficient without addressing structural barriers, such as poverty and limited access to food.

### 4.4 Limitations

This study has its limitations. First, the cross‐sectional design limits causal inference, making longitudinal studies necessary for a better understanding of the seasonal dynamics of food insecurity and dietary diversity. Second, reliance on self‐reported data (e.g., HFIAS, MAHFP) may introduce recall bias. Third, the sample of PLW (*n* = 642) for the dietary diversity analysis may not fully represent all women in the study area; therefore, further research should include non‐PLW to assess broader dietary patterns. Furthermore, the findings should be interpreted with caution, as several significant events occurring after data collection, such as prolonged droughts, armed conflicts, and locust invasions, may have further exacerbated food insecurity and dietary challenges in the study areas. These external shocks have the potential to undermine household resilience and intensify existing vulnerabilities, possibly altering the food security landscape since the data collection period.

## 5 Conclusion

In conclusion, this study highlights the widespread nature of food insecurity and low dietary diversity in the Seqota Declaration areas, driven by socio‐economic disparities, limited production diversity, and insufficient access to nutrient‐rich foods. Interventions should prioritize asset building, agricultural diversification, and equitable access to land to tackle these challenges. Future research should investigate longitudinal trends and the effectiveness of integrated nutrition and agricultural programs in enhancing food security and dietary outcomes in the Seqota Declaration woredas and rural Ethiopia.

## Author Contributions

**Andinet Abera Hailu:** conceptualization (equal), data curation (equal), formal analysis (equal), investigation (equal), methodology (equal), project administration (equal), software (equal), supervision (equal), validation (equal), visualization (equal), writing – original draft (equal), writing – review and editing (equal). **Stephen Thornhill:** conceptualization (equal), investigation (equal), visualization (equal), writing – review and editing (equal). **Edward Lahiff:** investigation (equal), methodology (equal), writing – review and editing (equal).

## Ethics Statement

The Ethiopian Public Health Institute (EPHI) Institutional Review Board reviewed and approved the study protocol, adhering to the principles outlined in the Declaration of Helsinki (World Medical Association 2013). All participants in the study were informed of their right to decline participation or to discontinue the interview at any time. Prior to the interview, written consent was obtained from each participant. All data collected during the study were securely stored on an institutional server.

## Consent

The authors have nothing to report.

## Conflicts of Interest

The authors declare no conflicts of interest.

## Data Availability

The primary data set collected from households and analyzed during the current study is available from the corresponding author.
